# The Medical Profession as a National Service

**Published:** 1918-11-09

**Authors:** 


### THE MEDICAL PROFESSION AS A NATIONAL SERVICE.

In the various discussions which relate to the future ordering of the national life there appears to be a general agreement that further steps must be taken to promote the well-being of the community by the promotion of the physical health of the individual citizen. Apart from other considerations, the statistics collected by the recruiting authorities have shown how large a proportion of the manhood of the nation falls below, and even seriously below, quite a moderate standard of health and efficiency. The figures are indeed impressive and it is recognised that they form a body of evidence which must weigh heavily in the development of any scheme of national reconstruction. To be added to this evidence is the practically universal admission that the existing arrangements for medical attendance on the poorer classes of the community during attacks of illness are incomplete and unsatisfactory. So soon as either one or the other of these two propositions comes under discussion there is inevitably raised a question of the position and work of the medical profession. Further, as it is allowed that both the raising of the standard of the general health of the nation, and an improvement in the amount and quality of the medical services available for the industrial and other classes of the community, involve conditions which can only be met by grants of public money, it necessarily follows that the relations of the State to the medical profession must be brought under review. Whatever scheme is adopted the doctors must be the agents for its application. But with public money paid to any individual or organisation goes necessarily more or less public control. Hence the State, or it may be the municipality, and the medical profession are necessarily brought face to face and the conditions of the relation must evidently be defined. Such is the position which the Government authorities, the public generally, and the medical profession are called upon to consider. All agree on the end to be sought. All agree that both the State and the profession have each a part to play in its accomplishment. It remains to find satisfactory terms on which this co-operation can be conducted.

With what may be called large schemes of preventive medicine there is little or no difficulty, at least from the medical point of view. The measures necessary for the avoidance of epidemic disease, the abolition in the home and in the community of conditions notoriously prejudicial to health, and undertakings closely related to these demands, manifestly require an elaborately organised Service conducted by officers who give their whole time to their official work. Here, plainly, the doctors are and must be State or municipal servants. Their duties are defined and ordered by a public authority, and by the same authority obviously their remuneration must be provided. In all this there is no contest. The arrangement already exists in the Public Health Service of the country, and no proposal to change it is under discussion. It may be enlarged or extended, but in essence it is accepted as coming under the general principle that public servants must be controlled by public authorities and paid out of public funds.

Far otherwise is the position when the subject of personal medical attendance on the sick individual comes into the debate. Until the date of the National Insurance Act every citizen in this respect acted for himself. He selected his own doctor as a matter of personal choice and paid the fee out of his own pocket, either directly or through the agency of a society or club organised for the purpose of meeting sickness on the principle of mutual insurance. The governing principle, in a word, was individual liberty. With the application of the National Insurance Act this position was necessarily modified. The individual citizen, if an employed person, was compelled to pay a weekly contribution and to select in advance as his medical adviser one or other of the doctors who elected to take service on the panel, and he could only alter his choice with difficulty. He paid something, it is true, but he did not pay all, for his employers, equally with himself, were compelled to a weekly contribution, and a further addition was made by the State. The individual citizen therefore lost some proportion of his liberty, and to compensate for this loss he was promised a more abundant and efficient medical service in times of illness. Whether this promise has been realised or not we need not now consider. Sufficient is it to say that on universal admission the medical services provided by the National Insurance Act do not satisfy present demands, and that on every side arises the claim that something better must be provided in the immediate future. If the scheme has not actually broken down it is certainly inadequate to the conditions which are presented as an essential part of any plan for national reconstruction. Hence the arrival of new proposals.

One of these new proposals is the organisation of the medical profession as a State Medical Service, and we print to-day a plan of this order by Colonel G. T. K. Maurice, C.M.G., together with the arguments which the author advances in its favour. For the moment we express no opinion on the matter. It is probable that we shall hear from members of the profession who are known to be strong opponents of the principle of a State Medical Service, and it may be our duty to sum up the discussion. Meanwhile, we commend Colonel Maurice's paper as a serious and thoughtful contribution to a difficult and important subject. When changes of one kind or another are recognised as inevitable there is no room for a mere arbitrary conservatism or for the closed mind. The time is one for an impartial consideration of the whole position and for an attentive hearing of all informed and honest workers. Criticism conducted in this spirit we shall welcome to our columns.

